# A frequent variant in the Japanese population determines quasi-Mendelian inheritance of rare retinal ciliopathy

**DOI:** 10.1038/s41467-019-10746-4

**Published:** 2019-06-28

**Authors:** Konstantinos Nikopoulos, Katarina Cisarova, Mathieu Quinodoz, Hanna Koskiniemi-Kuendig, Noriko Miyake, Pietro Farinelli, Atta Ur Rehman, Muhammad Imran Khan, Andrea Prunotto, Masato Akiyama, Yoichiro Kamatani, Chikashi Terao, Fuyuki Miya, Yasuhiro Ikeda, Shinji Ueno, Nobuo Fuse, Akira Murakami, Yuko Wada, Hiroko Terasaki, Koh-Hei Sonoda, Tatsuro Ishibashi, Michiaki Kubo, Frans P. M. Cremers, Zoltán Kutalik, Naomichi Matsumoto, Koji M. Nishiguchi, Toru Nakazawa, Carlo Rivolta

**Affiliations:** 1 Unit of Medical Genetics, Department of Computational Biology, University of Lausanne, 1015 Lausanne, Switzerland; 2 Service of Medical Genetics, Lausanne University Hospital (CHUV), 1011 Lausanne, Switzerland; 3 Department of Human Genetics, Yokohama City University Graduate School of Medicine, Yokohama, 236-0004 Japan; 4 Department of Human Genetics, Radboud University Medical Center, 6500 HB Nijmegen, The Netherlands; 5 Donders Institute for Brain, Cognition and Behaviour, Radboud University Nijmegen, 6525 GA Nijmegen, The Netherlands; 6 Laboratory for Statistical Analysis, RIKEN Center for Integrative Medical Sciences, Yokohama, 230-0045 Japan; 7 Department of Medical Science Mathematics, Medical Research Institute, Tokyo Medical and Dental University, Tokyo, 113-8510 Japan; 8 Department of Ophthalmology, Graduate School of Medical Sciences, Kyushu University, Fukuoka, 812-8582 Japan; 9 Department of Ophthalmology, Nagoya University Graduate School of Medicine, Nagoya, 466-8550 Japan; 10 Department of Integrative Genomics, Tohoku Medical Megabank Organization, Sendai, 980-8573 Japan; 11 Department of Ophthalmology, Juntendo University School of Medicine, Tokyo, 113-8421 Japan; 12 Yuko Wada Eye Clinic, Sendai, 980-0011 Japan; 13 RIKEN Center for Integrative Medical Sciences, Yokohama, 230-0045 Japan; 14 Institute of Social and Preventive Medicine, Lausanne University Hospital, 1011 Lausanne, Switzerland; 15 Department of Ophthalmology, Tohoku University Graduate School of Medicine, Sendai, 980-8574 Japan; 16 Department of Advanced Ophthalmic Medicine, Tohoku University Graduate School of Medicine, Sendai, 980-8574 Japan; 17 Department of Genetics and Genome Biology, University of Leicester, Leicester, LE1 7RH UK; 18 Institute of Molecular and Clinical Ophthalmology Basel (IOB), 4031 Basel, Switzerland; 19 University of Basel, 4001 Basel, Switzerland

**Keywords:** Medical genomics, Genetics of the nervous system, Hereditary eye disease

## Abstract

Hereditary retinal degenerations (HRDs) are Mendelian diseases characterized by progressive blindness and caused by ultra-rare mutations. In a genomic screen of 331 unrelated Japanese patients, we identify a disruptive *Alu* insertion and a nonsense variant (p.Arg1933*) in the ciliary gene *RP1*, neither of which are rare alleles in Japan. p.Arg1933* is almost polymorphic (frequency = 0.6%, amongst 12,000 individuals), does not cause disease in homozygosis or heterozygosis, and yet is significantly enriched in HRD patients (frequency = 2.1%, i.e., a 3.5-fold enrichment; *p*-value = 9.2 × 10^−5^). Familial co-segregation and association analyses show that p.Arg1933* can act as a Mendelian mutation in *trans* with the *Alu* insertion, but might also associate with disease in combination with two alleles in the *EYS* gene in a non-Mendelian pattern of heredity. Our results suggest that rare conditions such as HRDs can be paradoxically determined by relatively common variants, following a quasi-Mendelian model linking monogenic and complex inheritance.

## Abstract (web summary)

Genetic variants in *RP1* can cause hereditary retinal degeneration (HRD). Here, in a genomic screen of 331 Japanese HRD patients, the authors identify a near-polymorphic *RP1* variant that causes Mendelian HRD in *trans* with an *Alu* insertion and otherwise is associated with HRD according to a complex model of inheritance.

## Introduction

Together with intellectual disabilities, hereditary retinal degenerations (HRDs, comprising retinitis pigmentosa and allied diseases) represent a group of conditions for which both genetic and allelic heterogeneity is the highest in humans^1,2^. To date, almost 300 genes and thousands of mutations have been identified as causative of HRD, and the detection of novel disease genes and variants continues at a steady pace (RetNet database: https://sph.uth.edu/retnet/). Considering that the overall prevalence of HRDs does not exceed 1 in 2000 individuals, the average contribution of any given HRD gene to the disease is incredibly small. Similarly, apart from two DNA variants that appear to be relatively frequent in the general population and determine a specific form of the disease (p.Asn1868Ile and p.Gly863Ala in *ABCA4*)^3,4^, most mutations are so rare that they are seldom detected in more than one pedigree, worldwide. In addition, although HRDs affect people from the five continents, their specific allelic assortment seems to be population-specific^5,6^. For instance, similar to other islanders or groups of people that have experienced relative historical isolation, Japanese carry certain alleles, including pathogenic ones, which are not found elsewhere in the world^7^. Furthermore, lack of significant reduction in fitness before the reproductive age, associated with such an elevated heterogeneity, have led to the consequence that the number of recessive mutations that are detected heterozygously in the general, unaffected population is remarkably high and may affect up to one person in two^8^.

Despite this extraordinary variability and abundance of mutations, HRDs are almost invariantly inherited as a monogenic, Mendelian trait, for which the presence of only one (dominant) or two (recessive) mutations in the same gene, genome-wide, is at the same time a necessary and sufficient condition for pathogenicity^9^. At the other end of the spectrum of ocular conditions having a genetic component lies age-related macular degeneration (AMD), another retinal disease affecting people aged 50 and over. AMD is a bona fide complex disease with a relatively high prevalence (1 in 13 individuals), favored by the presence of polymorphic SNPs, highly penetrant rare variants, and environmental factors^10^. Between these two pillars of inheritance, there is an intermediate zone, consisting in a few examples for which extremely rare mutations in more than one gene are associated with Bardet–Biedl syndrome, a retinal ciliopathy displaying sometimes digenic triallelic inheritance^11–13^.

*RP1* is one of the several HRD genes identified to date, and one of the few causing disease by more than one Mendelian pattern of inheritance. Originally described as linked to autosomal dominant retinitis pigmentosa (adRP)^14–16^, a subtype of HRD, it was later shown to be associated with a recessive form of the same disease (arRP)^17^. To date, at least 60 mutations have been reported in *RP1*, most of which cluster within its last exon (exon 4), cumulatively accounting approximately for 5.5% and up to 4.5% of all adRP and arRP cases, respectively^18,19^. However, some DNA variants at the far 3’ end of the gene, including nonsense variants, appear not to cause disease, at least not according to a dominant or recessive pattern of inheritance^20,21^. *RP1* encodes a multi-modular protein of 2156 amino acids, which is a member of the doublecortin family and is present in the ciliary axoneme of both rods and cones, the light-sensing neurons of the retina^22,23^. Mutations in *RP1* thus determine visual loss as a consequence of a ciliopathic phenotype affecting these specialized cell types.

Following the screen of a large set of Japanese HRD patients, we identify three mutations in the *RP1* gene: a mobile *Alu* element insertion in exon 4, a novel frameshift mutation, and a nonsense variant in the far 3’ part of the coding sequence. While the first two variants behave as classical recessive Mendelian alleles, NM_006269.1:c.5797C>T/p.Arg1933* appears to cause disease according to a more complex pattern of inheritance. When present in *trans* with respect to the *Alu* insertion, it acts as a Mendelian mutation. Furthermore, despite being enriched in patients vs. controls, p.Arg1933* is completely benign in homozygosis or in heterozygosis. By performing an association test between 28 HRD patients, heterozygous carriers of this nonsense allele, and 3554 controls, we find that p.Arg1933* may be pathogenic not only as a Mendelian allele, but also in association with variants elsewhere in the genome, and in particular with two DNA changes in another ciliary gene, *EYS*.

## Results

### An *Alu* insertion in *RP1* is a prevalent cause of HRD in Japan

**Fig. 1:**
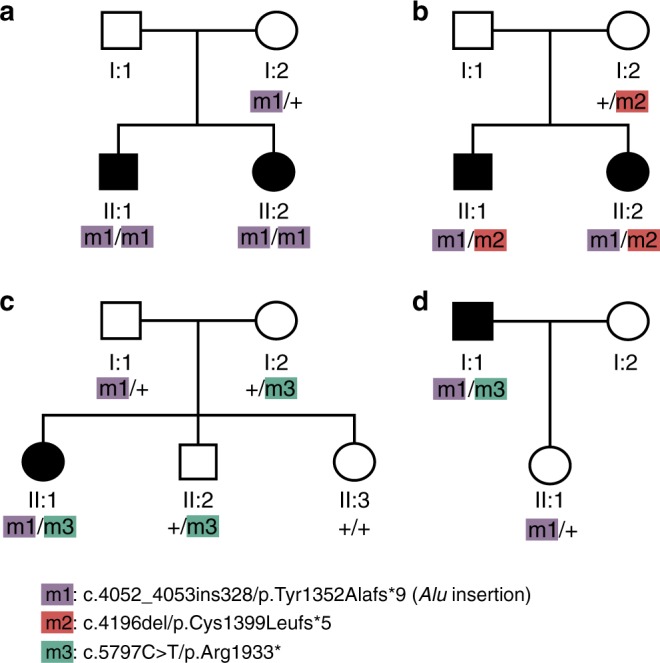
Segregation analysis of the *RP1* mutations found in this study. Pedigrees of representative families are shown

In the framework of a screening effort of 331 unrelated Japanese patients, we identified a novel, unusual mutation by whole-genome sequencing, consisting in the insertion of a mobile *Alu* element in exon 4 of the *RP1* gene (m1, or NM_006269.1:c.4052_4053ins328/p.Tyr1352Alafs*9) in a female individual from a recessive HRD family. This insertion caused the disruption of the reading frame by introducing 328 additional nucleotides, including a premature termination codon in the canonical *RP1* coding sequence. The mother of the proband was heterozygous for this variant and the proband’s affected brother was also a homozygote, in support of the notion that this was indeed a recessive HRD mutation (Fig. 1a). Targeted screening for this *Alu* insertion in the remaining 330 patients (all forms of HRDs, isolate or recessive cases, not genetically pre-screened), as well as in 524 Japanese controls, available for direct testing of m1, identified 15 other affected and unrelated individuals and one heterozygous control carrying this insertion. In total, six patients were homozygous for the mutation (12 alleles), which co-segregated with the disease as a classical Mendelian, recessive allele, whenever this could be tested, while 10 carried it heterozygously. Altogether, these findings indicate that this *Alu* insertion is not only clearly pathogenic, but it is also a rather prevalent cause of retinal degeneration within the Japanese islands at the level of a single allele (1.8% of all HRD Japanese patients), possibly second only to the most frequent mutation so far identified in this country, i.e., NM_001142800.1:c.4957dup in *EYS*^24–26^.

Remarkably, 6 of the 10 individuals who carried the *Alu* insertion heterozygously were in fact compound heterozygotes for either of two other changes in *RP1*: a novel frameshift mutation (c.4196del/p.Cys1399Leufs*5, m2, two unrelated individuals) and a nonsense variant c.5797 C > T/p.Arg1933* (m3, four unrelated individuals) that was previously identified in the general population and is present in dbSNP as entry #rs118031911 (Supplementary Table 1). Again, both variants, detected by direct Sanger sequencing, co-segregated with the disease in relevant families, according to an autosomal recessive pattern of inheritance (Fig. 1b–d).

### m3 is enriched in patients, but does cause HRD per se

Frameshift c.4196del/p.Cys1399Leufs*5 (m2) was absent from 3480 Japanese control chromosomes and was reported in the gnomAD database^27^ to have an allele frequency of 5.44 × 10^−5^ in East Asia, indicating that this DNA variant is a very rare allele, as it is the case for most HRD mutations.

In contrast, the rs118031911/T allele (m3), despite being virtually absent in many world populations, was found to be relatively frequent in East Asians (Supplementary Fig. 1), and probably too frequent to be a Mendelian allele for HRD, according to the Hardy–Weinberg model. In particular, our direct screening of 12,379 Japanese individuals with no retinal degeneration showed the presence of rs118031911/T in 145 subjects, 142 heterozygotes and 3 homozygotes (148 alleles), validating the notion that this DNA variant is in fact almost polymorphic in Japan (allele frequency = 0.6%). All these subjects were examined by fundoscopy and, in addition, we evaluated clinically one of the three homozygotes (the only one who could be re-assessed, in agreement with our Institutional Review Board protocol) by a very thorough ophthalmological examination. At age 28 years old, she had no visual symptoms and displayed no ocular abnormalities: she had normal visual acuity (20/20 in both eyes), intact visual field (Goldmann perimetry), and no evidence of retinal degeneration through slit lamp examination and fundoscopy. Furthermore, optical coherence tomography imaging, used to assess detailed retinal structures, showed no signs of retinal thinning and electroretinogram, a test allowing objective detection of minimal retinal dysfunction even in the absence of subjective symptoms, showed normal responses. Finally, absence of late-onset HRD, who could have escaped detection in a 28-year-old individual, was confirmed by the assessment of the fundi of the other two rs118031911/T homozygotes, who displayed no signs of retinal degeneration at ages of 78 and 79 years, respectively. Overall, both population based-data and direct clinical assessments confirm that rs118031911/T does not cause HRD per se, in heterozygosis or in homozygosis.

However, specific screening for the rs118031911/T allele in the same cohort of 331 Japanese HRD patients mentioned above led to the identification of 10 additional heterozygotes (14 alleles in total) showing that its frequency in HRD patients was 2.1% (14 alleles out of 662) (Supplementary Fig. 1). The 3.5-fold enrichment of rs118031911/T in patients vs. controls (148 alleles out of 24,758 = 0.6%) was highly significant [*p*-value = 9.2 × 10^−5^, threshold = 0.05/*N*, *N* = 1, by Fisher’s exact test (24,610:148 vs. 648:14)], indicating that this relatively common variant has in fact an effect on retinal health. WES analysis of these 10 patients detected no mutations in HRD genes that could explain their phenotype, according to a Mendelian fashion of inheritance. Considering that rs118031911/T introduces a nonsense codon in the *RP1* open reading frame and was found in *trans* with respect to the *Alu* insertion in some patients, it is not unlikely that it could represent a hypomorphic variant contributing to the mutational load of genes involved in retinal homeostasis. In other words, despite being benign when considered as a Mendelian allele (monoallelically or biallelically), rs118031911/T could exert a pathogenic function in conjunction with DNA changes in other known HRD genes, according to an oligogenic pattern of inheritance that was previously modeled for hereditary ciliopathies^28–30^.

### A non-Mendelian pattern of inheritance for m3

**Fig. 2:**
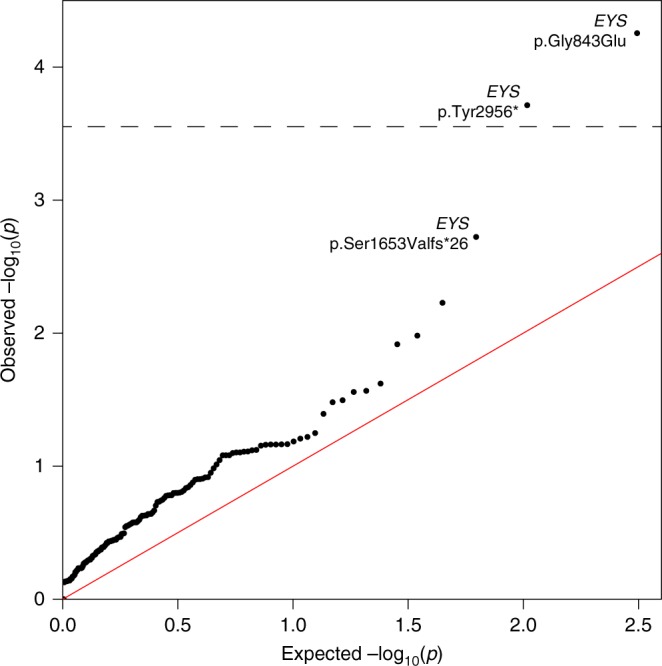
Results of the association study. Quantile–quantile (Q–Q) plot of rare/low-frequency non-synonymous variants in HRD genes in 28 patients heterozygous for rs118031911/T vs. 3554 Japanese controls. The significance threshold is indicated by the dotted line

We tested this hypothesis by assessing enrichment of nonsynonymous, rare, and low-frequency variants (minor allele frequency between 0.1% and 5%, according to published literature; further details in Methods) in m3 carriers. We analyzed the 10 patients mentioned above, as well as 18 additional patients with the same genotype (heterozygous for rs118031911/T, with no other recognized mutations in *RP1* and no mutations in HRD genes that could explain their phenotype), identified following a targeted screening of 713 Japanese HRD cases from another internal cohort (Supplementary Table 1). Specifically, we performed an association test between these 28 individuals and 3554 Japanese controls from the 3.5KJPN database^31^ by considering all 228 bona fide HRD genes^32^ from the RetNet database (Supplementary Table 2) that could produce multiallelic inheritance of HRD in m3 heterozygotes, in line with previous protocols involving similar analyses^33–38^. Cryptic relatedness among patients, as well as the presence of additional, undetected *RP1* mutations in *trans* with respect to rs118031911/T were excluded prior to performing the test (Supplementary Table 3 and Supplementary Data 1). The association analysis identified two variants that were significantly enriched in patients vs. controls (Table 1, Fig. 2). Interestingly, although they were not in linkage disequilibrium, both variants belonged to the gene *EYS* (NM_001142800.1:c.2528 G > A;p.Gly843Glu and c.8805 C > A;p.Tyr2935*, *p*-values = 5.6 × 10^−5^ and 1.9 × 10^−4^, respectively; threshold = 2.8 × 10^−4^ = 0.05/*N*, where *N* = 178, by Fisher’s exact test), possibly highlighting a mechanism of pathogenesis directly involving the proteins EYS and RP1. Indeed, a third DNA change within *EYS* (c.4957del;p.Ser1653Valfs*26) ranked 3rd in the list of associated variants, even if its *p*-value did not reach statistical significance after Bonferroni correction. Furthermore, we performed the same analysis by considering not only variants from RetNet sequences, but from the whole human exome, in both cohorts. In support of the data obtained, the two significant hits detected in the HRD gene set were also the two top hits detected exome-wide, even though no variant reached the threshold for exome-wide significance. Altogether, these results indicate that the rs118031911/T nonsense could act in concert with at least two DNA changes (and possibly with more) to determine a pathological phenotype in a non-Mendelian fashion.

**Table 1:** Summary of the results of the association study

Gene	Variant	Frequency in cases	Frequency in controls	OR	95% CI (OR)	*p*-value
*EYS*	NM_001142800.1:c.2528G>A; p.Gly843Glu	0.125	0.017	8.23	3.08–18.78	5.6E-05*
*EYS*	NM_001142800.1:c.8805C>A; p.Tyr2935*	0.054	0.002	33.30	5.86–128.76	1.9E-04*
*EYS*	NM_001142800.1:c.4957del; p.Ser1653Valfs*26	0.054	0.004	13.85	2.62–46.83	1.9E-03
*USH2A*	NM_206933.2:c.15355C>T; p.Arg5119Trp	0.036	0.002	20.15	2.16–92.44	5.9E-03
*USH2A*	NM_206933.2:c.2802T>G; p.Cys934Trp	0.036	0.003	14.55	1.60–63.22	0.010
*PDZD7*	NM_024895.4:c.1267G>A; p.Ala423Thr	0.036	0.003	13.38	1.48–57.70	0.012
*EYS*	NM_001142800.1:c.7394C>G; p.Thr2465Ser	0.089	0.029	3.28	1.01–8.30	0.024
*CC2D2A*	NM_001080522.2:c.501G>T; p.Lys167Asn	0.036	0.004	8.44	0.96–34.65	0.027
*RPGRIP1L*	NM_015272.4:c.171G>T; p.Leu57Phe	0.054	0.011	4.90	0.96–15.63	0.028
*BBIP1*	NM_001243783.2:c.112T>C; p.Ser38Pro	0.036	0.005	7.70	0.87–31.35	0.032

*Note*: The top 10 hits from this test are shown

*OR* odds ratio, *CI* confidence interval

*p-values retaining statistical significance

Based on previous data on Bardet-Biedl syndrome^11^, we tested a digenic diallelic vs. triallelic mode of action for rs118031911/T on HRD, by comparing the frequency of this variant in patients for whom the molecular causes of retinal degenerations were identified (i.e., solved cases) vs. unsolved HRD cases vs. controls. As expected, a comparison of unsolved vs. controls showed significance, as reported above, whereas solved vs. controls did not show any significant enrichment for rs118031911/T (6 rs118031911/T variants over 722 alleles for solved vs. 148 over 24,758 for controls, *p*-value = 0.46, OR = 1.4, CI = 0.50–3.14, by Fisher’s exact test). Comparison of solved vs. unsolved HRD cases showed borderline non-significant enrichment for rs118031911/T in unsolved cases (*p*-value = 0.07, OR = 2.30, CI = 0.94–6.76, by Fisher’s exact test), possibly indicating that either well-defined triallelism does not take place for this variant or that we did not have enough power to detect it.

## Discussion

The extreme genetic heterogeneity of retinal degenerations, together with the elevated number of pathogenic and hypomorphic changes in HRD genes that are detected in the unaffected population, have evoked the theoretical possibility that non-Mendelian, oligogenic inheritance could be responsible for these conditions^9^. Digenic heredity has been clearly demonstrated for specific combinations of mutations^39–41^ in particular pedigrees or in individual cases, including digenic triallelic transmission of Bardet–Biedl syndrome^11,42^. For these patients, the presence of two (diallelic) or three (triallelic) mutations at two different loci (digenism) causes disease, presumably by compromising the overall function of gene products that belong to the same complex or are part of the same biochemical pathway. This model seems to be particularly true for genes encoding for proteins that form or play a role within the cell primary cilium, according to the paradigm of mutational load put forward by N. Katsanis and coworkers^43^. In these instances, accumulation of rare variants (which individually may have a little effect) in multiple ciliary genes can produce a pathological phenotype that is connected to ciliary function and result in a ciliopathy, including retinal ciliopathies^44–46^.

Intriguingly, despite our association test was not limited to ciliary genes, both our significant hits lie within a ciliary gene, *EYS*. In primates, the EYS protein has been shown to physically co-localize with RP1 in the ciliary axoneme of photoreceptors and is thought to play a role in the structural organization and maintenance of these cells’ apical part, the outer segment (OS)^47^. This functional role is further supported by studies in zebrafish, where *EYS* knockouts show progressive retinal degeneration due to mis-localization of specific OS proteins and the disruption of F-actin filaments^48,49^, a key component not only for the integrity, but also for the morphogenesis of the OS^50^. In a similar fashion, targeted disruption of the *RP1* gene in mice leads to defects of the OS, because of the incorrect stacking of its discs^51^. The co-localization of RP1 and EYS, as well as their common role in the homeostasis of the OS, strongly indicates that they may have synergic functions and that pathogenesis could occur in a digenic fashion.

In this work we show that two specific *RP1* alleles are responsible for a relatively large number of Mendelian HRD cases in Japan. Interestingly, none of these two changes is a rare allele at all, compared to the average frequencies of classical HRD mutations. The first, the c.4052_4053ins328/p.Tyr1352Alafs*9 *Alu* element insertion in *RP1* (m1), seems to be the second most common HRD recessive mutation described so far in Japan, and its frequency may even be underestimated, since insertional events of mobile elements are difficult to detect by conventional screening techniques. The second variant, c.5797 C > T/p.Arg1933* or rs118031911/T (m3), is even more frequent, and by far more interesting. Despite introducing a premature stop codon in the *RP1* open reading frame, this DNA change is almost polymorphic in East Asia and does not cause disease either in heterozygous or homozygous carriers. However, this same change may act as pathogenic allele in a Mendelian fashion (with another *RP1* mutation in *trans*), or in association with rare variants in at least another gene, according to a non-Mendelian, possibly oligogenic pattern of inheritance. Although we currently ignore the molecular mechanisms leading to this unusual model of pathogenicity, it is probably the consequence of an increased global mutational load with threshold effect, determined by the accumulation of variants with different pathogenic potential. The presence of one or of two rs118031911/T alleles likely produces a load that is below this pathological threshold, while the co-occurrence of extra variants could result in the crossing of such a limit for normal retinal homeostasis. This hypothesis is supported by the evidence that rs118031911/T is pathogenic in conjunction with a very severe mutation, i.e., the insertion of an *Alu* element in *RP1*’s exon 4 mentioned above, which completely ablates the open reading frame of the gene. We term this model of inheritance quasi-Mendelian, to define the differential behavior (Mendelian or non-Mendelian) that specific alleles may have with respect to different genotypes at the same locus or elsewhere in the genome.

**Fig. 3:**
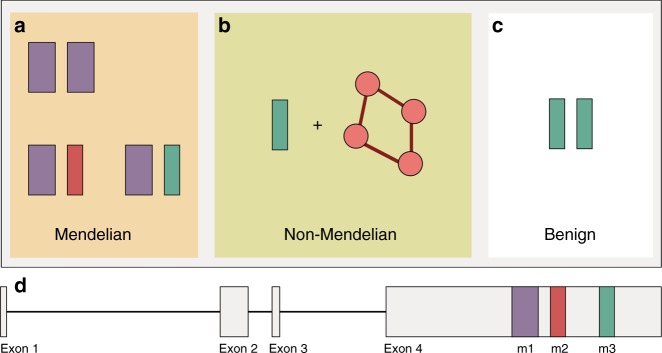
Schematic representation of the inheritance pattern of the identified mutations in *RP1*, highlighting the concept of rs118031911/T-mediated quasi-Mendelian inheritance of HRDs. **a** In *trans* with respect to the *Alu* element insertion (m1, or c.4052_4053ins328/p.Tyr1352Alafs*9), m3 (rs118031911/T, or c.5797C>T/p.Arg1933*) results in autosomal recessive inheritance of the disease, similar to m1 in a homozygous state or in a compound heterozygous combination with m2 (c.4196del/p.Cys1399Leufs*5). **b** Combinations of the hypomorphic m3 allele with additional hypomorphs and/or heterozygous recessive alleles in other genes result in disease following a non-Mendelian pattern, whereas (**c**) homozygosis for m3 has no pathological consequences. **d** Structure of *RP1*: exons are represented by boxes, connected by solid lines (introns). The relative positions of m1, m2, and m3 are also indicated

In conclusion, it seems that, at least for *RP1*-associated HRD, disorders displaying a Mendelian pattern of inheritance may also genetically behave like multigenic conditions, for which both polymorphic (having a low effect) and rare (having a rather high effect) variants can determine pathogenesis (Fig. 3). The findings from our study require further replication, ideally in other East Asian cohorts. However, the low prevalence of HRD and the even lower percentage of HRD patients carrying rs118031911/T limit our ability to assemble cohorts of sufficient power at this time, which would not only enable us to validate our findings but also to propose more defined models of pathogenicity. To better illustrate this: the identification of the 28 heterozygotes reported in this study corresponds to the screening of roughly 5–10 million Japanese individuals. Nevertheless, our work provides a proof of concept that a non-negligible proportion of HRDs can be caused by inheritance mechanisms that transcend the Mendelian model, to be investigated in detail by future, very large-scale and population-specific sequencing endeavors, such as for instance the 100,000 genomes project^52^. Furthermore, our findings suggest that oligogenic heredity of human diseases (and perhaps of other traits) may not be limited to a low number of cases with ultra-rare conditions, as shown up to now^34,35,53,54^, but could extend to more frequent phenotypes and represent a bridge between monogenic and complex inheritance.

## Methods

### Subjects

The study was initiated following the approval by the Institutional Review Boards of our respective Institutions (University of Lausanne, Yokohama City University Graduate School of Medicine, Radboud University Medical Center, Kyushu University, Nagoya University Graduate School of Medicine, Tohoku Medical Megabank Organization, Juntendo University School of Medicine, Tohoku University Graduate School of Medicine, and Tokyo Medical and Dental University). All subjects provided written informed consent, and the study was conducted in adherence with the Declaration of Helsinki.

Tohoku University School of Medicine, Kyushu University School of Medicine, and Nagoya University School of Medicine, all based in Japan, were the centers where all Japanese patients with HRD were recruited. HRD was diagnosed clinically after excluding possible secondary causes of retinal degeneration such as toxicity and uveitis. Final diagnosis required the presence of reduced electroretinogram (ERG) responses, visual field loss, and funduscopic abnormalities consistent with retinal degeneration (retinal vascular narrowing and abnormalities of the retinal pigment epithelium etc.) symmetrically in both eyes.

Genotypes from individuals without HRD were collected from both published and unpublished databases [the BioBank Japan Project (*N* = 12,379), the ToMMo Japanese Reference Panel Project (3.5KJPN release, *N* = 3554)^31^, the Tohoku University School of Medicine (*N* = 95), the Yokohama City University Graduate School of Medicine (*N* = 429)] or were obtained experimentally by direct genotyping of genomic DNA, with standard molecular biology techniques.

Summary phenotypes of carriers of m1, m2, or m3 mutations are listed in Supplementary Table 1.

### Whole genome sequencing and analysis

Genome sequencing of the first index patient was performed using the sequencing platform by Complete Genomics^55^. Sequence reads were mapped to the human reference genome (NCBI build 37) and variants were called genome-wide. These included: single-nucleotide variants (SNVs), copy-number variations (CNVs), as well as structural variations (SVs) such as *Alu* element insertions and/or chromosomal rearrangements. Data were extracted from MasterVar files and other relevant matrices by ad hoc Perl, bash, and R scripts, available upon request. Assessment of pathogenic variants was performed as previously described^56^.

### Screening for the *Alu* element insertion

In order to screen for the presence of the *Alu* element in exon 4 of *RP1* distinct pair of primers were designed (forward: 5′-AGGCTTGTTTCCTAGGAGAGGT-3′, reverse: 5′-TTCTGCTTCTTTTTCACTTAGGC-3′) using the CLCbio Genomics Workbench (Qiagen, Hilden, Germany).

PCR amplification was performed in a 20 μl total volume containing 20 ng genomic DNA, 1× GoTaq buffer, 0.5 mM dNTPs, 10 μM of each primer, and 2 units (5 U/μl) of GoTaq polymerase (Promega, Madison, Wisconsin). PCR products were separated following agarose gel electrophoresis. PCR products displaying abnormal size profiles were purified (ExoSAP-IT, USB, Cleveland Ohio) and a sequencing reaction was performed in a total volume of 5 μl using 1 μl primer 3.3 µM, 0.5 μl BigDye Terminator v1.1, and 1 μl of the provided Buffer (Applied Biosystems, Foster City, California) Big Dye terminator cycle sequencing kit on an ABI 3130xl Genetic Analyzer (Applied Biosystems).

For this screening, we used 524 controls from the Tohoku University School of Medicine (*N* = 95) and the Yokohama City University Graduate School of Medicine (*N* = 429).

### Whole exome sequencing and analysis

Paired-end DNA sequencing libraries of 28 individuals were generated using Aglilent SureSelect Human All ExonV6 kit (Agilent Technologies, CA, USA) by Novogene Co., Ltd., Hong Kong. One microgram of genomic DNA per sample was fragmented into 180–280 bp fragments by hydrodynamic shearing (Covaris, Massachusetts, USA). After the reparation of the 3’ and 5’ ends and the adenylation of the 3’ ends, paired-end adaptors were ligated to the DNA fragments. DNA fragments with ligated adaptors on both ends were enriched by PCR. PCR products were further purified using the AMPure XP system (Beckman Coulter, Beverly, USA) and quantified using the Agilent high sensitivity DNA assay on the Agilent Bioanalyzer 2100 system. Captured DNA fragments were sequenced on an Illumina NovaSeq 6000 platform (Illumina, San Diego, California).

Raw sequence files were assessed, trimmed, and mapped to the human genome reference sequence (UCSC hg19) using Novoalign V3.08.02 (Novocraft, Selangor, Malaysia). Variants were called jointly by GATK 3.8^57^ and annotation was performed using EPACTS (http://genome.sph.umich.edu/wiki/EPACTS) and Annovar^58^. The nomenclature of all variants studied was validated by using VariantValidator^59^.

All single nucleotide variants were further filtered to obtain only high-quality variants. Briefly, quality control was carried out using the following parameters: (1) remove individual calls if Depth (DP) < 8 or GenotypeQuality (GQ) < 20, (2) exclude variants if the average GQ value ≤35, (3) exclude variants if call-rate value ≤ 0.9, (4) keep only variants with no deviation from Hardy-Weinberg equilibrium (*p* > 0.05 after Bonferroni correction), (5) keep variants passing GATK VQSR (VQSRTranche of 90.0), (6) final hard filtering step with Quality by Depth (QD) ≥ 2, FisherStrand (FS) ≤ 60, RMSMappingQuality (MQ) ≥ 40, MappingQualityRankSumTest (MQRankSum) ≥ −12.5, ReadPosRankSumTest (ReadPosRankSum) ≥ −8, StrandOddsRatio (SOR) ≤ 3 and ExcessHet ≤ 20.

### Association study on rare and low-frequency variants in RetNet genes

An association test was performed on rare and low-frequency non-synonymous variants from a curated RetNet list (*N* = 228 genes, Suppl. Table 2) to test possible association of variants in the 28 patients carrying rs118031911/T heterozygously compared to 3554 controls from the ToMMo database^31^. More specifically, variants were retained if they had a frequency comprised between 0.1 and 5% in controls, with the exclusion of rs118031911/T itself, according to published methods^60–62^, and in particular to the paper by Marouli and coworkers^60^. This resulted in the selection of 178 variants in 84 different genes, which were used to test association in rs118031911/T carriers vs. controls by Fisher’s exact test with an experiment-wide Bonferroni-corrected threshold of 2.81 × 10^−4^, for α = 0.05 (0.05/178 = 2.81 × 10^−4^). *P*-values and odds ratios were obtained by the fisher.test function with default parameters in R (v3.5.1) and the Q–Q plot (Fig. 2) was obtained by using the qqman package^63^.

### Relatedness analyses

PLINK (v1.90b5)^64^ was used to compute PI_HAT values between all pairs of the 28 rs118031911/T carriers using calls from whole exome sequencing. This analysis showed no relatedness, with PI_HAT values between 0.00 and 0.07 (threshold for relatedness = 0.2)^65^, for all 378 possible pairwise combinations (Supplementary Data 1).

### SNP genotyping of the *RP1* locus

To detect haplotypes in *trans* with respect to rs118031911/T, we genotyped 23 SNPs encompassing the *RP1* locus over ~260 kb, by standard techniques.

## Supplementary information

Supplementary Information

Supplementary Data 1

Supplementary Data 2

Description of Additional Supplementary Files

## Data Availability

The data supporting the findings of this study, as a whole, contain information that could compromise the privacy/consent of the participants, therefore we provide the genotypes of the cases analyzed as a summary statistics file containing aggregated data (Supplementary Data 2). Genotypes of control Japanese individuals from the ToMMo Japanese Reference Panel Project (3.5KJPN release, v20181105open) can be accessed at https://jmorp.megabank.tohoku.ac.jp/201905/downloads. Data from the BioBank Japan Project and the Tohoku University School of Medicine and Yokohama City University Graduate School of Medicine is available upon request to the corresponding author, pending authorization of the Centers that generated them and in agreement with their specific IRB approvals.

## Code availability

Processing of raw sequence files, variant calling, variant annotation, as well as all the other analyses described in this work were performed using available and open source software, as detailed in the Methods section. No custom code was therefore used, aside from simple scripts integrating commands for such programs.
