# Supplementary material for: A frequent variant in the Japanese population determines quasi-Mendelian inheritance of rare retinal ciliopathy
Source: Nat Commun. 2019 Jun 28;10:2884. doi: 10.1038/s41467-019-10746-4 (PMC6599023; doi:10.1038/s41467-019-10746-4)
Supplement: Supplementary file 1 — Supplementary Information [file 41467_2019_10746_MOESM1_ESM.pdf]

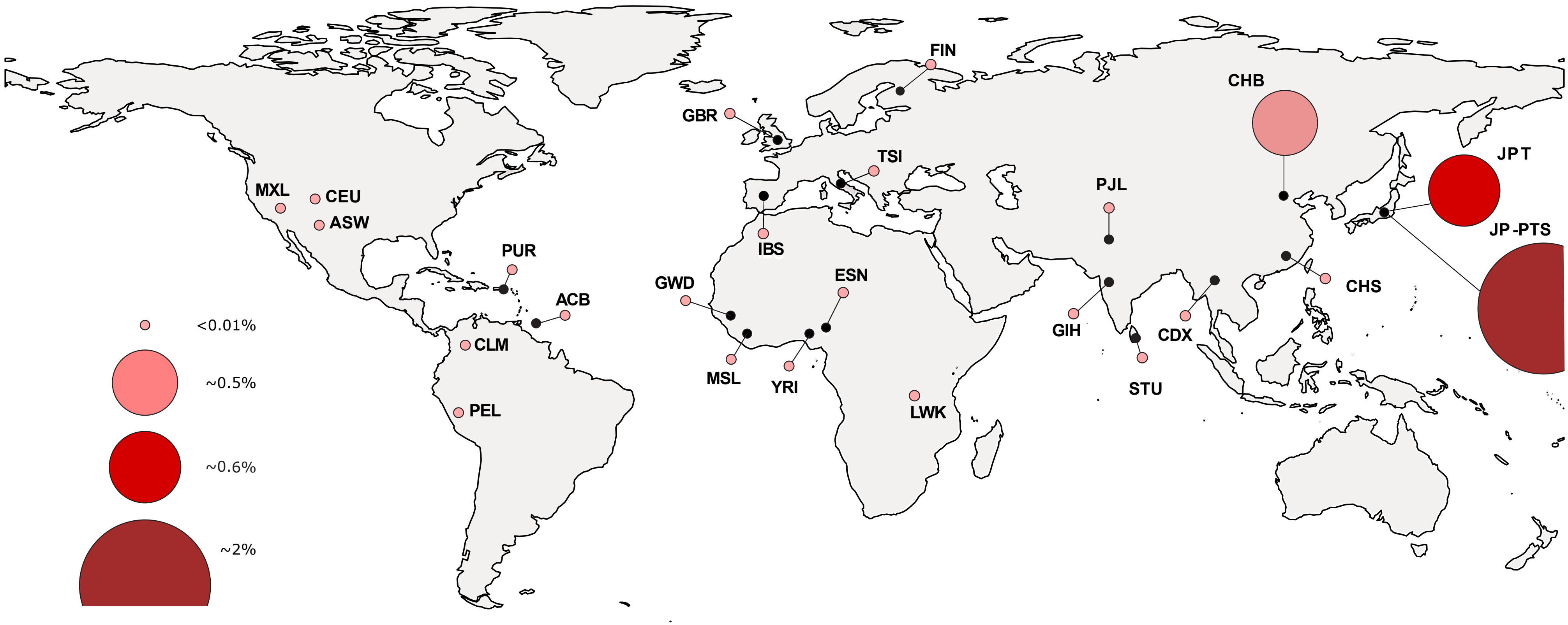

Supplementary Figure 1. Frequency of the rs118031911/T allele across different world populations. ACB, African Caribbeans in Barbados; ASW, Americans of African Ancestry in South West USA; BEB, Bengali from Bangladesh; CDX, Chinese Dai in Xishuangbanna, China; CEU, Utah Residents (CEPH) with Northern and Western European Ancestry; CHB, Han Chinese in Bejing, China; CHS, Southern Han Chinese; CLM, Colombians from Medellin, Colombia; ESN, Esan in Nigeria; FIN, Finnish in Finland; GBR, British in England and Scotland; GIH, Gujarati Indian from Houston, Texas; GWD, Gambian in Western Divisions in the Gambia; IBS, Iberian Population in Spain; JPT, Japanese in Tokyo, Japan; JP-PTS, Japanese HRD patients from our set; LWK, Luhya in Webuye, Kenya; MSL, Mende in Sierra Leone; MXL, Mexican Ancestry from Los Angeles USA; PEL, Peruvians from Lima, Peru; PJL, Punjabi from Lahore, Pakistan; PUR, Puerto Ricans from Puerto Rico; STU, Sri Lankan Tamil from the UK; TSI, Toscani in Italia; YRI, Yoruba in Ibadan, Nigeria. This figure was generated in part by using the 'maps' package for R, version 3.3.0.

Supplementary Table 1. Clinical data for patients heterozygous for m3 or with biallelic m1, m2, or m3 mutations. MD, macular dystrophy; CD, cone dystrophy; CRD cone-rod dystrophy; RP, retinitis pigmentosa.

Patient ID	Diagnosis	Age at examination	Gender	Age of symptoms onset	Genotype
M15	MD	73	M	53	m3/+
M9	CD	84	F	60	m3/+
R(O)40	RP	37	F	elementary school	m3/+
R21	RP	63	M	unknown	m3/+
221	RP	67	M	~30	m3/+
R(O)44	RP	30	F	18	m3/+
OPH-610	RP	48	F	20	m3/+
OPH-302	RP central type	37	F	childhood	m3/+
Q-116	RP	60	F	childhood	m3/+
OPH-179	RP	78	M	20	m3/+
OPH-285	RP	55	F	40	m3/+
OPH-419	RP	44	F	18	m3/+
OPH-424	RP	84	M	10	m3/+
OPH-733	RP	68	M	50	m3/+
OPH-794	RP	50	M	40	m3/+
OPH-812	RP	80	M	26	m3/+
OPH-814	CRD	51	F	20	m3/+
OPH-884	RP	58	F	40	m3/+
OPH-967	RP	37	M	25	m3/+
R170	RP	31	M	31	m3/+
R204	RP	47	F	4	m3/+
YWC101	RP	41	M	8	m3/+
YWC102	RP	44	M	30	m3/+
YWC107	RP	73	F	65	m3/+
OPH-553	RP	60	M	40	m3/+
YWC100	RP	42	F	20	m3/+
YWC193	RP	69	F	20	m3/+
YWC6	RP	42	F	35	m3/+
R(O)39	RP	29	F	elementary school	m1/m1
R(O)48	RP	12	F	12	m1/m1
R(O)70	RP	34	F	elementary school	m1/m1
RN36	RP	32	M	6	m1/m1
OPH-635	RP	46	M	12	m1/m1
08_20	RP	54	F	30s	m1/m1
201	RP	28	F	5	m1/m2
209	RP	26	F	childhood	m1/m2
C1	MD	41	F	25	m1/m3
M5	MD	55	M	44	m1/m3
M7	MD	43	F	36	m1/m3
OPH-280	RP	61	F	50	m1/m3

Supplementary Table 2. HRD genes (from RetNet) used for the association test (N=228).

ABCA4	CEP164	GPR179	MKKS	PRPF3	SNRNP200
ABCC6	CEP250	GRK1	MKS1	PRPF31	SPATA7
ABHD12	CEP290	GRM6	MVK	PRPF4	SPP2
ACBD5	CEP78	GUCA1A	MYO7A	PRPF6	TIMM8A
ADAM9	CERKL	GUCA1B	NBAS	PRPF8	TIMP3
ADAMTS18	CFAP410	GUCY2D	NDP	PRPH2	TMEM216
ADGRA3	CHM	HARS	NEK2	PRPS1	TMEM237
ADGRV1	CIB2	HGSNAT	NMNAT1	RAB28	TOPORS
AGBL5	CLN3	HK1	NPHP1	RAX2	TRIM32
AHI1	CLRN1	HMX1	NPHP3	RBP3	TRNT1
AHR	CLUAP1	IDH3A	NPHP4	RBP4	TRPM1
AIPL1	CNGA1	IDH3B	NR2E3	RCBTB1	TTC8
ALMS1	CNGA3	IFT140	NRL	RD3	TTLL5
ARHGEF18	CNGB1	IFT172	NYX	RDH11	TTPA
ARL2BP	CNGB3	IFT27	OAT	RDH12	TUB
ARL3	CNNM4	IFT81	OFD1	RDH5	TUBGCP4
ARL6	CRB1	IMPDH1	OPN1LW	REEP6	TUBGCP6
ARSG	CRX	IMPG1	OPN1MW	RGR	TULP1
ASRGL1	CSPP1	IMPG2	PANK2	RGS9	USH1C
ATF6	CTNNA1	INPP5E	PCARE	RGS9BP	USH1G
BBIP1	CWC27	INVS	PCDH15	RHO	USH2A
BBS1	CYP4V2	IQCB1	PCYT1A	RIMS1	WDPCP
BBS10	DHDDS	ITM2B	PDE6A	RLBP1	WDR19
BBS12	DHX38	JAG1	PDE6B	ROM1	WFS1
BBS2	DRAM2	KCNJ13	PDE6C	RP1	WHRN
BBS4	DTHD1	KCNV2	PDE6G	RP1L1	ZNF408
BBS5	EFEMP1	KIAA1549	PDE6H	RP2	ZNF423
BBS7	ELOVL4	KIF11	PDZD7	RP9	ZNF513
BBS9	EMC1	KIZ	PEX1	RPE65	
BEST1	EXOSC2	KLHL7	PEX2	RPGR	
C1QTNF5	EYS	LAMA1	PEX7	RPGRIP1	
C8orf37	FAM161A	LCA5	PHYH	RPGRIP1L	
CABP4	FLVCR1	LRAT	PITPNM3	RS1	
CACNA1F	FZD4	LRIT3	PLK4	SAG	
CACNA2D4	GDF6	LRP5	PNPLA6	SAMD11	
CC2D2A	GNAT1	LZTFL1	POC1B	SDCCAG8	
CCT2	GNAT2	MAK	POC5	SEMA4A	
CDH23	GNB3	MERTK	POMGNT1	SLC24A1	
CDH3	GNPTG	MFRP	PRCD	SLC25A46	
CDHR1	GPR125	MFSD8	PROM1	SLC7A14	

Supplementary Table 3. Haplotype surrounding rs118031911/T (in bold) across ~260 kb, in 11 randomly-selected patients out of the 28 p.Arg1933* heterozygotes considered for the association test. Positions are given with respect to the hg19 genome build.

Chromosome	Position	Change	OPH-967	YWC101	YWC102	YWC107	OPH-179	OPH-812	OPH-794	OPH-884	OPH-814	OPH-419	OPH-424
8	55505788	A>G	G	G	G	A	G	G	G	G	G	G	G
8	55506499	T>G	T	T	T	T	T	T	T	T	T	G	G
8	55513201	A>G	A	G	A	A	A	A	G	A	A	G	A
8	55522880	G>A	G	A	G	G	G	G	A	G	G	G	G
8	55523753	A>G	A	A	A	A	A	A	A	A	A	G	A
8	55529612	T>C	C	T	C	T	T	C	T	T	C	T	C
8	55539057	C>T	T	C	T	C	C	T	C	C	T	C	C
8	55539395	T>A	T	T	T	T	T	T	T	T	T	A	T
8	55541450	C>T	T	C	T	C	C	T	C	C	T	C	C
8	55541513	A>G	G	A	G	A	A	G	A	A	G	A	A
**8**	**55542239**	**C>T**	**T**	**T**	**T**	**T**	**T**	**T**	**T**	**T**	**T**	**T**	**T**
8	55542540	C>T	C	C	C	C	C	C	C	C	C	T	C
8	55552310	T>C	T	T	T	T	T	T	T	T	T	C	T
8	55629852	A>G	A	G	A	G	G	A	G	G	A	A	G
8	55632762	G>A	G	A	G	A	A	G	A	A	G	G	A
8	55678538	T>G	T	G	T	G	G	T	G	G	T	T	G
8	55688171	G>A	G	A	G	A	A	G	A	A	G	G	G
8	55692780	A>G	A	G	A	G	G	A	G	G	A	A	G
8	55693133	C>T	C	C	C	C	C	C	C	C	C	T	C
8	55735722	C>T	C	T	C	T	C	T	T	T	C	C	C
8	55756256	G>A	G	G	G	G	G	G	G	G	G	A	G
8	55761124	A>G	A	G	A	G	G	A	G	G	A	G	G
8	55763773	G>A	G	A	G	A	A	G	A	A	G	G	G
