# Supplementary material for: A frequent variant in the Japanese population determines quasi-Mendelian inheritance of rare retinal ciliopathy
Source: Nat Commun. 2019 Jun 28;10:2884. doi: 10.1038/s41467-019-10746-4 (PMC6599023; doi:10.1038/s41467-019-10746-4)
Supplement: Supplementary file 4 — Description of Additional Supplementary Files [file 41467_2019_10746_MOESM4_ESM.docx]

**Description of additional Supplementary Files**

**Title: Supplementary Data 1
Description:** PI_HAT values for the 28 RP1 m3 carriers.

**Title: Supplementary Data 2
Description:** Aggregated genotypes of the 28 RP1 m3 carriers.
